# *In Vitro* Screening for Antihepatic Steatosis Active Components within Coptidis Rhizoma Alkaloids Extract Using Liver Cell Extraction with HPLC Analysis and a Free Fatty Acid-Induced Hepatic Steatosis HepG2 Cell Assay

**DOI:** 10.1155/2013/459390

**Published:** 2013-12-18

**Authors:** Hui Fan, Yuan-yuan Chen, Wei-jian Bei, Lai-you Wang, Bao-tian Chen, Jiao Guo

**Affiliations:** ^1^Key Unit of Modulating Liver to Treat Hyperlipemia SATCM (State Administration of Traditional Chinese Medicine), SATCM Level 3 Lab of Lipid Metabolism, Guangdong TCM Key Laboratory for Metabolic Diseases, Institute of Chinese Medicinal Sciences, Guangdong Pharmaceutical University, Guangzhou Higher Education Mega Centre, Guangzhou 510006, China; ^2^College of TCM, Southern Medical University, Guangzhou 510515, China

## Abstract

A high-throughput method was developed and applied to screen for the active antihepatic steatosis components within Coptidis Rhizoma Alkaloids Extract (CAE). This method was a combination of two previously described assays: HepG2 cell extraction with HPLC analysis and a free fatty acid-induced (FFA) hepatic steatosis HepG2 cell assay. Two alkaloids within CAE, berberine and coptisine, were identified by HepG2 cell extraction with HPLC analysis as high affinity components for HepG2. These alkaloids were also determined to be active and potent compounds capable of lowering triglyceride (TG) accumulation in the FFA-induced hepatic steatosis HepG2 cell assay. This remarkable inhibition of TG accumulation (*P* < 0.01) by berberine and coptisine occurred at concentrations of 0.2 **μ**g/mL and 5.0 **μ**g/mL, respectively. At these concentrations, the effect seen was similar to that of a CAE at 100.0 **μ**g/mL. Another five alkaloids within CAE, palmatine, epiberberine, jateorhizine, columbamine, and magnoline, were found to have a lower affinity for cellular components from HepG2 cells and a lower inhibition of TG accumulation. The finding of two potent and active compounds within CAE indicates that the screening method we developed is a feasible, rapid, and useful tool for studying traditional Chinese medicines (TCMs) in treating hepatic steatosis.

## 1. Introduction

Hepatic steatosis, a condition characterized by excessive fat accumulation within hepatocytes, is becoming a serious global health threat. Despite this, there are currently no effective treatments available for this condition [1, 2]. Traditional Chinese medicines (TCMs) serve as an excellent alternative and/or complementary treatment for hepatic steatosis [3]. Previous work in China studying extracts classified as TCMs has found them effective as antihepatic steatosis agents [4–9]. However, due to the uncertainty concerning the active components of these extracts as well as the possible role of multiple components, clinical application has been limited [10]. Therefore, determining the active ingredients within these clinically applicable extracts is an important avenue of study.

In order to determine the active components against hepatic steatosis within these TCMs extracts, a high-throughput screening protocol was developed. One method within this protocol involves using cell extraction of the component of interest combined with HPLC analysis to identify which component from multicomponent materials has an affinity for cellular components [11]. This method is based on the affinity of the active component for the living cell, where the active components have the highest affinity for the cells and, therefore, can be extracted after the drug has been incubated with the cell line. The general procedure for cell extraction with HPLC analysis is as follows: the cells are cultured, the drug is incubated with the cells, an elution is performed to remove any thing binding with low specificity binding, and a targeted extraction is done for high-affinity binding to cellular components followed by identification by HPLC analysis. This method has previously been successfully applied to screen active components from TCMs [12, 13].

Another screening assay available for determining antihepatic steatosis agents uses an *in vitro* free fatty-acid- (FFA-) induced hepatic steatosis HepG2 cell model. Oil Red O staining and intracellular triglyceride (TG) contents are used in this assay to evaluate the activity of the compounds of interest on lowering the lipid levels within the cells. This method has been used previously to validate the activity of components in TCMs [14, 15].

In this study, these two assays, HepG2 cell extraction with HPLC analysis and FFA-induced hepatic steatosis in HepG2 cells, were combined to develop a new method to screen for the antihepatic steatosis active components from Coptidis Rhizoma alkaloid extracts (CAE). The HepG2 cell extraction with HPLC analysis was used to screen for CAE components with high-affinity for hepatocytes. Then, the FFA-induced hepatic steatosis HepG2 cell assay verified the antisteatosis activity of the identified components.

Recent studies using high-fat feed induced hyperlipidemia animal models revealed that CAE may reduce total cholesterol (TC) and triglyceride (TG) levels [16]. Also, CAE has a hepatoprotective effect on CCl_4_ induced acute liver injuries [17, 18]. These studies suggest that CAE has significant hepatoprotective effects and can be used to treat hepatic steatosis. However, CAE is composed of multiple components [19], predominantly several alkaloids: berberine, palmatine, jateorhizine, epiberberine, coptisine, columbamine, and magnoflorine. Presently, only berberine, the alkaloid present in the highest amount in CAE, has been paid much attention, whereas other alkaloids remain unstudied. We therefore used our screening method in order to explore the possible antihepatic steatosis active components in CAE.

## 2. Material and Reagents

**Figure 1:**
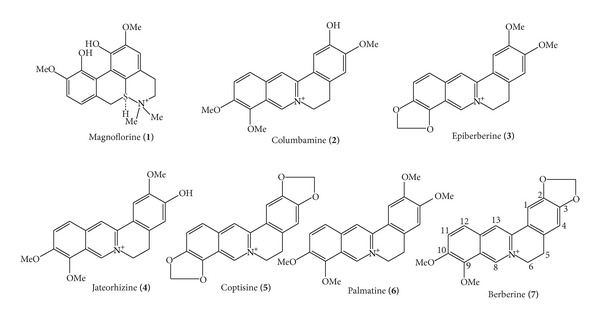
The chemical structure of seven alkaloids.

Berberine, palmatine, coptisine, jateorhizine, and magnoflorine (Figure 1), purity of >98%, were purchased from Chengdu Herbpurify Co., LTD (Chengdu, China) for use as standards. Epiberberine and columbamine were supplied by the Chongqing Academy of Chinese Materia Medica. Sodium oleic and sodium palmitic were purchased from Sigma (Madrid, Spain). HPLC-grade acetonitrile was purchased from Honeywell International Inc. (Burdick & Jackson, Muskegon, MI, USA). Deionized water was purified using a PURELAB Ultra GE MK2 water system (ELGA, High Wycombe, UK). DMEM medium was from Gibco. Triglyceride enzymatic assay kits were from Nanjing Jiancheng Bioengineering Institute (Nanjing, China). All other reagents used were of analytical grade at minimum. Samples of Coptidis Rhizoma were provided by the Zhixin Chinese Herbal Medicine Co., Ltd. (Guangzhou, China) and authenticated by Professor Shuyuan Li, pharmacognosist of the School of Chinese Medicinal Sciences, Guangdong Pharmaceutical University.

## 3. Methods 

### 3.1. The Preparation and Chromatographic Analysis of CAE

To prepare the CAE, a method mimicking the TCM approach was used [20]. Briefly, the dry rhizomes were cut into pieces, and 100 g was added to 600 mL of 70% ethanol and immersed for 30 min at 25°C. This mixture was heated under reflux for 120 min. Following filtration, the extraction was repeated on the residue twice for a total of three times. The three extract solutions were then combined and evaporated until the volume was 75 mL under reduced pressure. The alcohol extract was reconstituted with 1% acetic acid, adjusted to a pH of 3.0 using 1.0 mmol/L HCl and salted by adding in 6% NaCl. The precipitate from this step was dried at 40°C under a DZF-6021 vacuum drying oven (Hangzhou Lihui Environmental Testing Equipment Co. Ltd., Hangzhou, China).

For the chromatographic analysis, the HPLC system Dionex UltiMate 3000 (Dionex, Germany) used came equipped with Chromeleon software (Dionex) and was comprised of a quaternary pump, an online vacuum degasser, an autosampler, a thermostated column compartment, and DAD. All separations were carried out on a DIONEX Acclaim C_18_ column (250 mm × 4.6 mm, 5.0 *μ*m) with the column temperature maintained at 30°C. The isocratic mobile phase was used and consisted of acetonitrile-potassium dihydrogen phosphate solution (0.015 mol/L) (40/60, v/v) (1.7 g/L sodium dodecyl sulfate, phosphoric acid to adjust to pH 3.0) pumped at a flow rate of 1.0 mL/min. The injection volume was 10 *μ*L and the detection wavelength was 270 nm.

### 3.2. Cell Culture

HepG2 cells, a human hepatoblastoma cell line, were cultured at 37°C in a humidified 5% CO_2_ atmosphere in high glucose Dulbecco's modified Eagle's medium (DMEM) supplemented with 10% fetal bovine serum (FBS) and an 1% antibiotic mixture of penicillin (100 U/mL) and streptomycin (100 mg/mL). This media was changed every 2 days until the cells were 70% confluent, usually 5–7 days after initial seeding. Prior to each experiment, the cells were cultured in the absence of FBS for 24 h.

### 3.3. Liver Cell Extraction

HepG2 cells were grown in a cell culture flask for at least 24 h prior to treatment with alkaloids. Once the HepG2 cells were 70% confluent, they were treated with a working solution of different alkaloids or DMEM alone as a negative control at 37°C for 24 h. A cell suspension was made from each experimental flask and was centrifuged at 210 ×g for 10 min. The supernatant was then removed and the remaining pellet was washed five times with 2 mL of PBS (pH 7.4) followed by centrifugation at 210 ×g for 10 min to remove any compounds binding with low affinity. The last one of PBS washes was collected and used as a low specificity reference for HPLC analysis. Finally, the cell pellet was lysed by adding 2 mL of 75% ethanol and repeatedly freeze-thawed. This solution was centrifuged at 8000 ×g for 10 min and the supernatant was filtered through a 0.22 *μ*m membrane and analyzed by HPLC.

CAE was used at a working concentration of 100.0 *μ*g/mL and incubated with HepG2 cells following the cell viability assay. A mixed standard working solution composed of the seven alkaloids in equal concentrations was prepared and incubated with HepG2 cells at 10 *μ*g/mL. In addition, gemfibrozil, a lipid regulator, was used as a positive control for liver cell extraction at a working concentration of 100.0 *μ*g/mL as previously described [21].

### 3.4. Cell Viability Assay

FFA-induced cytotoxicity of HepG2 cells was assessed by the (MTT) assay in the presence or absence of the alkaloids. After incubating for 24 h, 20 *μ*L of MTT solution was added to each well and the plates were further incubated at 37°C for 4 h. The media was then removed, 100 *μ*L of DMSO was added and the plates were gently shaken for 5 min. An enzyme-linked immunosorbent assay was performed and the optical absorbance was determined at 485 nm (Mithras LB 940, Berthold Technologies, Germany). Each condition was performed in triplicate.

### 3.5. *In Vitro* Antihepatic Steatosis Assay

HepG2 cells were seeded in 6-well plates at a density of 2 × 10^5^ cells in 2.0 mL/well of 10% FBS-DMEM medium and incubated for 48 h. Upon reaching 80–90% confluency, the cultured cells were incubated with 0.5 mmol/L FFA (sodium oleic/sodium palmitic, 2 : 1) plus the different alkaloid solutions for 24 h. The alkaloid solutions were applied to the cells at different concentrations and each sample was treated with preventive administration in the medium. One control consisted of cells exposed to the 0.5 mmol/L FFA in media in the absence of additional alkaloids or CAE and was designated the “model group.” Another control consisted of cells treated with FFA-free medium. There were 6 parallel holes at every group.

All alkaloid reference standards were dissolved in DMSO. The appropriate concentrations of CAE were prepared in 0.1% hydrochloric acid. In order to investigate the dose-dependent effect of alkaloids on antihepatic steatosis, a series of concentrations of each alkaloid and the CAE were prepared.

### 3.6. Intracellular TG Content and Oil Red O Staining

The amount of lipid accumulation in HepG2 cells was investigated by measuring TG content and Oil Red O staining. Intracellular TG content was determined using the EnzyChrom triglyceride assay kit according to the manufacturer's instructions and was normalized to the total protein content of each experimental sample. Protein content was measured using the BCA Protein Assay Kit (Cwbiotech, China) according to the manufacturer's instructions. TG accumulation inhibition rates were calculated using the following equation:

(1) TG  accumulation  on⁡  inhibition  rate  (%) =((TG  contents  of  model  group  −TG  content  of  sample  group)×(TG  content  of  model  group)−1)×100.

The Oil Red O staining was performed by fixing the samples in 4% paraformaldehyde and then staining with Oil Red O for 15 min. The samples were washed with isopropanol for a few seconds, followed by three distilled water washes. Results were determined by inverted fluorescence microscopy (HAL-100 Zeiss, Germany).

### 3.7. Statistical Analysis

The data is displayed as mean ± SD. Statistical significance between each experimental group was determined by Student's test using SPSS software (version 16.0), where *P* < 0.05 was considered statistically significant.

## 4. Results

### 4.1. The Chromatographic Analysis of CAE

**Figure 2:**
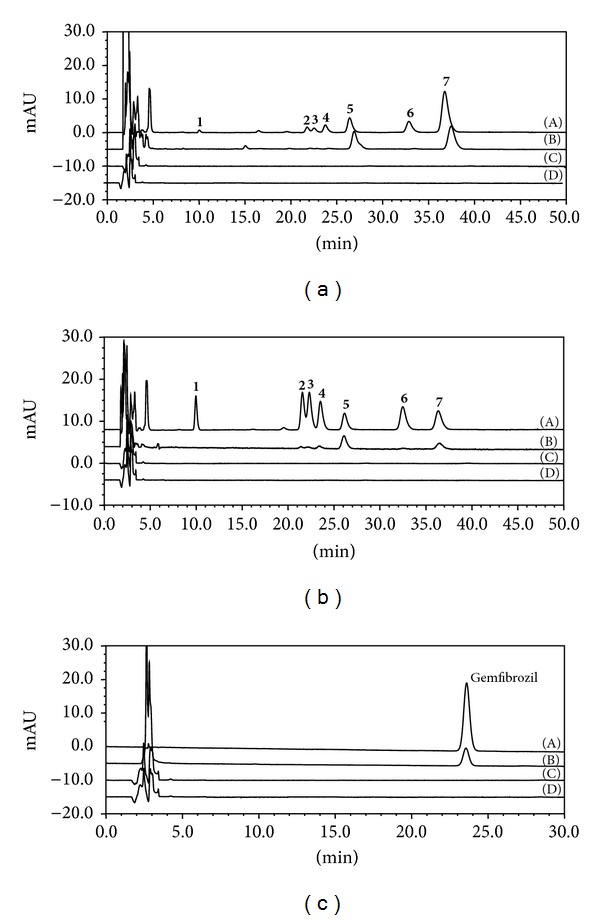
The HPLC chromatograms of HepG2 cell extracts analyzed at 270 nm following treatment with (a) CAE, (b) a mixed standard of seven alkaloids, or (c) gemfibrozil. The lines for ((A)–(D)) represent the (A) sample work solution, (B) the denatured desorption elute of HepG2 cells incubated with sample, (C) the final washing elute of HepG2 cells incubated with sample, and (D) the denatured desorption eluate of HepG2 cells cultured in medium without sample (blank). Peaks identified as (**1**) magnoflorine, (**2**) columbamine, (**3**) epiberberine, (**4**) jateorhizine, (**5**) coptisine, (**6**) palmatine, and (**7**) berberine.

The chromatographic parameters were optimized to achieve high resolution of all seven alkaloids in the CAE solution. The seven alkaloids were able to be clearly separated and detected by UV detector at 270 nm as shown in Figure 2(a)-Line (A). The reference standards were used to quantify each alkaloid within CAE at the 100.0 *μ*g/mL concentration. The data showed that the alkaloid present at the highest amount was berberine at 31.74 *μ*g/mL. The next most abundant were coptisine and palmatine at 10.64 *μ*g/mL and 9.10 *μ*g/mL, respectively. The other alkaloids were present at lower concentrations with epiberberine at 4.56 *μ*g/mL, columbamine at 1.44 *μ*g/mL, magnoflorine at 1.30 *μ*g/mL, and jateorhizine at 1.10 *μ*g/mL. The comparison of the chromatogram of CAE in Figure 2(a)-Line (A) and the mixed reference standard in Figure 2(b)-Line (A) indicated that the seven alkaloids are the major peaks seen in CAE and make up 59.88% of the CAE solution.

### 4.2. Cytotoxic Effect on HepG2 Cells

HepG2 cells were treated with 0–2.0 mmol/L FFA mixture (sodium oleic/sodium palmitic, 2 : 1) for 24 h and the FFA-induced cytotoxicity of HepG2 cells was measured by MTT assay. FFA was not cytotoxic at concentrations lower than 1.0 mmol/L. The cytotoxicity of different concentrations of the alkaloids and CAE combined with FFA (0.5 mmol/L) were subsequently measured by MTT assay. It was found that CAE had no toxicity on HepG2 cells in the tested concentrations of up to 100.0 *μ*g/mL. Also, there were no cytotoxic effects of each alkaloid on HepG2 cells at concentrations of less than 50.0 *μ*g/mL during 24 h incubation.

### 4.3. Liver Cell Extraction

Chromatograms of CAE liver cell extractions are shown in Figure 2(a). There were two peaks detected by HPLC at 270 nm in the extract of denatured HepG2 cells (Figure 2(a)-Line (B)). By comparing each peak's retention time with the corresponding standard (Figure 2(b)-Line (B)), peaks 5 and 7 were identified as coptisine at 26.4 min and berberine at 36.8 min. The chromatogram of the low-specificity elution was used (Figure 2(a)-Line (C)) to discount any confounding peaks that the eluting process may produce. Meanwhile, the blank liver cell extraction without added CAE or alkaloids was performed to discount any peaks from the cell components themselves (Figure 2(a)-Line (D)). The peaks that were determined to be from CAE or the alkaloids were absent from both the low-specificity elution and the blank cell extraction as berberine and coptisine are the two most abundant components of CAE. The next step was to explore whether the affinity of the tested compounds for the cells was related to the concentration of each component. Therefore, the mixed reference alkaloid solution was prepared containing the same concentration of each alkaloid, 10.0 *μ*g/mL, and was used to treat HepG2 cells as previously described. Using this reference solution, we confirmed that berberine and coptisine, the same high-affinity components previously identified, were extracted from denatured HepG2 cells (Figure 2(b)-Line (B)). This suggests there is no correlation between the concentration of the substance and its binding affinity. Additionally, the peak areas ratio of berberine to coptisine following extraction was 1.22 higher than the 0.72 before extraction. It was suggested that the binding ability of coptisine to HepG2 cells is possibly stronger than that of berberine, regardless of the concentration of berberine in CAE. Berberine and coptisine were defined as components capable of binding with high-affinity to liver cells, while the other five alkaloids can bind with low affinity. The gemfibrozil used as a positive control demonstrated that the extraction from HepG2 cells was successful (Figure 2(c)).

### 4.4. FFA-Induced Hepatic Steatosis HepG2 Cells Model

A hallmark of hepatic steatosis is abnormal TG accumulation within liver cells. An *in vitro* FFA-induced hepatic steatosis HepG2 cell assay has been previously employed and acknowledged as an effective screening model. FFA consisting of oleic acid/palmitic acid added exogenously at a 2 : 1 ratio has been reported to be optimum for inducing this condition [22]. In order to increase the solubility of oleic acid and palmitic acid in the growth medium, the FFA mixture of sodium oleic/sodium palmitic at a 2 : 1 ratio was used and then the concentration was further optimized. It was found that FFA treatment at concentrations from 0.1 to 2.0 mmol/L for 24 h resulted in a dose-dependent intracellular TG level increase that was significant (*P* < 0.01) up to 0.5 mmol/L FFA as compared to the control group. When administering the combination of alkaloids, the concentration of 0.5 mmol/L FFA (sodium oleic/sodium palmitic at 2 : 1) was decided on as the induction concentration of the model group. Oil Red O staining (Figure 5(b)) shows that lipid droplets accumulated significantly in the HepG2 cell cytoplasm at 0.5 mmol/L FFA.

### 4.5. TG Reduction by CAE and Alkaloids

First, a series of CAE concentrations was used to treat the hepatic steatosis HepG2 cell model for 24 h. It was found that CAE treatment at concentrations ranging from 12.5 *μ*g/mL to 100.0 *μ*g/mL had a TG reducing effect in a dose-dependent manner, whereas 50.0 *μ*g/mL achieved the significant inhibitory effect (*P* < 0.01) shown in Figure 4. This indicated that CAE had anti-TG accumulation activity.

**Figure 3:**
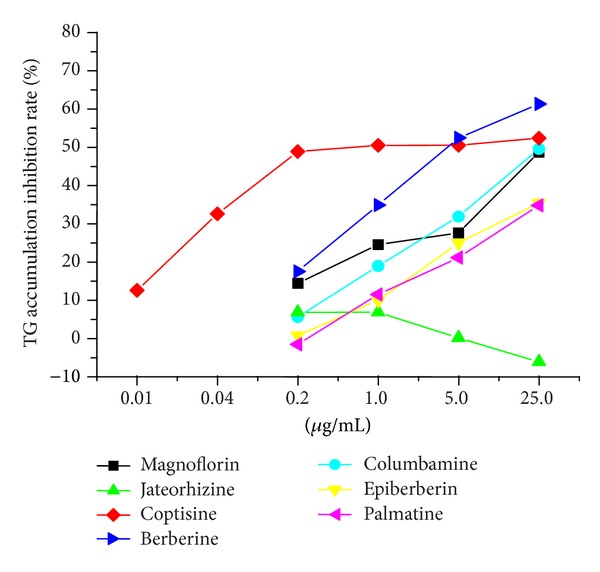
The trend chart of the rate of TG accumulation inhibition following treatment with alkaloids at different concentrations on a hepatic steatosis HepG2 cell model.

We previously found that seven alkaloids make up the main marker peaks in chromatographic analysis of CAE. Therefore, the TG reduction ability of every alkaloid was evaluated using a series of concentrations for each alkaloid. The concentrations of each alkaloid were determined based on their relative content within 100.0 *μ*g/mL CAE. Each alkaloid was applied at concentrations of 0.2, 1.0, 5.0, and 25.0 *μ*g/mL, and the inhibition of TG accumulation was evaluated.  Table 1 shows the TG inhibition activity of each alkaloid concentration series. Several major results were found. (1) Berberine exhibited a potent inhibitory effect on the intracellular TG levels in a dose-dependent manner at 0.2 *μ*g/mL with a significance of *P* < 0.05 and 1.0 *μ*g/mL with a significance of *P* < 0.01. The highest concentration tested, 25.0 *μ*g/mL, had a remarkable ability to inhibit 61.3% of TG accumulation. (2) Coptisine demonstrated the strongest inhibitory effect on the intracellular TG levels; however, there was no dose-dependent effect seen from concentrations of 0.2 *μ*g/mL to 25 *μ*g/mL. At a 0.2 *μ*g/mL concentration, coptisine already significantly (*P* < 0.01) reduced TG accumulation by 48.9%. However, a dose-dependent effect was seen at the lower end of the concentration gradient, 0.01 *μ*g/mL (*P* < 0.05) to 0.2 *μ*g/mL (*P* < 0.01), during additional treatments. (3) Although palmatine, columbamine, epiberberine, and magnoflorine also exhibited a dose-dependent relationship with intracellular TG level reduction, it was only at the highest concentration, 5.0 *μ*g/mL, that this reduction was significant (*P* < 0.01). (4) Jateorhizine failed to inhibit the FFA-induced hepatic steatosis model at concentrations ranging from 0.2 *μ*g/mL to 25.0 *μ*g/mL and had no dose-dependent effect. To better understand the inhibition activity of the seven alkaloids, the TG inhibition rate for each alkaloid at each concentration tested was expressed as a trend graph (Figure 3). At a 5.0 *μ*g/mL concentration, the alkaloids ware differentiated into two groups: a high activity group consisting of coptisine and berberine, and a low activity group consisting of the rest of the alkaloids. The inhibition by coptisine was more potent than berberine at lower concentrations ranging from 0.2 *μ*g/mL to 1.0 *μ*g/mL. Compared to coptisine and berberine, the other alkaloids tested, not including jateorhizine, showed a weaker inhibition of TG accumulation. Jateorhizine failed to measurably inhibit TG accumulation.

### 4.6. TG Reduction Capabilities of Alkaloid Combinations

**Figure 4:**
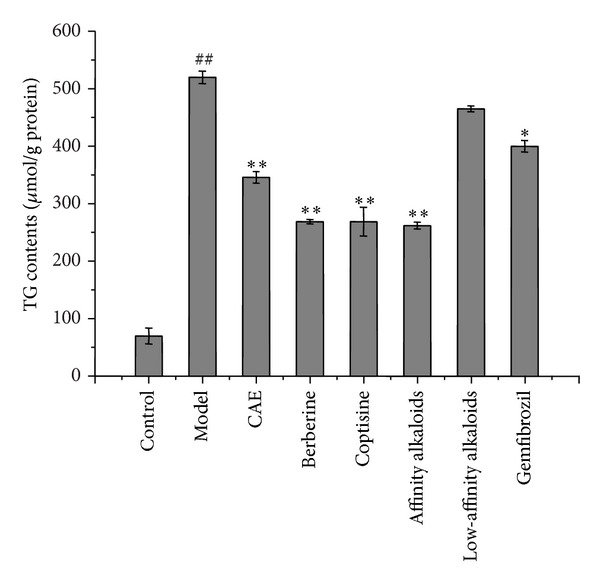
The combinatorial analysis of seven alkaloids: 50.0 *μ*g/mL CAE, 15.87 *μ*g/mL of berberine, 5.32 *μ*g/mL of coptisine, a high-affinity alkaloid group composed of 15.87 *μ*g/mL berberine, 5.32 *μ*g/mL coptisine, a low-affinity alkaloid group composed of 4.55 *μ*g/mL palmatine, 2.28 *μ*g/mL epiberberine, 0.55 *μ*g/mL jateorhizine, 0.72 *μ*g/mL columbamine, 0.65 *μ*g/mL magnoline, and 100.0 *μ*g/mL gemfibrozil. The selected concentration of alkaloids was based on the concentration ratio within 50.0 *μ*g/mL CAE.

**Figure 5:**
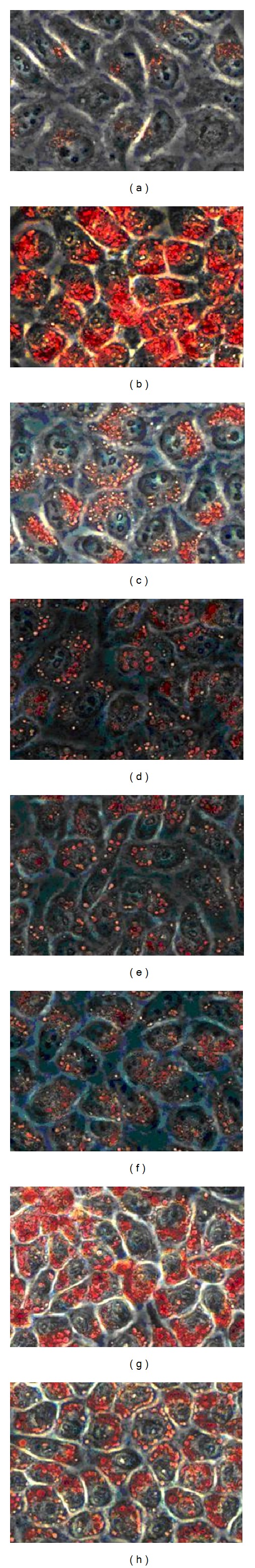
The Oil Red O staining (400x) effect of combinatorial treatment: (a) control cells, (b) 0.5 mmol/L FFA-induced steatosis model cells, (c) 50.0 *μ*g/mL CAE, (d) 15.87 *μ*g/mL berberine, (e) 5.32 *μ*g/mL coptisine, (f) a high-affinity alkaloid group composed of 15.87 *μ*g/mL berberine and 5.32 *μ*g/mL coptisine, (g) a low-affinity alkaloid group composed of 4.55 *μ*g/mL palmatine, 2.28 *μ*g/mL epiberberine, 0.55 *μ*g/mL jateorhizine, 0.72 *μ*g/mL columbamine, and 0.65 *μ*g/mL magnoline, and (h) 100.0 *μ*g/mL gemfibrozil.

A combinatorial analysis was carried out to further verify the TG accumulation inhibition activity of coptisine and berberine, as well as evaluate whether the alkaloids have any synergistic or antagonistic effects with one another. The coptisine and berberine were combined to make a high-affinity alkaloid group, while the other five alkaloids were combined to make a low-affinity alkaloid group at concentrations based on their abundance in 50.0 *μ*g/mL CAE. The TG reduction results are shown in Figure 4. The high-affinity alkaloid group had a potent inhibitory effect on TG reduction (*P* < 0.01), whereas the low-affinity alkaloid group still showed no significant inhibition. Compared with coptisine and berberine alone, the combination coptisine and berberine group had no increase in TG reduction, indicating there are no synergistic effects by these alkaloids on TG accumulation. Supporting these results, Oil Red O staining (Figure 5) showed that the lipid droplets were decreased significantly in the high activity component treatment groups, including CAE, berberine, coptisine, and the combination coptisine and berberine group, whereas the low-affinity alkaloid combined group showed a weak effect.

## 5. Discussions

The metabolism of oral drugs starts with absorption into the bloodstream, where the drugs are maintained in their ingested form or metabolized and then delivered into organs and cells. In this study, a comprehensive pharmacokinetic study was undertaken. Prototype alkaloids, including jateorhizine, berberine, coptisine, and palmatine have previously been identified and quantified in rat plasma following oral administration by Wuji Pill using LC-MS/MS [23]. Further investigation by Xiexin Decoction in rats found that three of these, alkaloids, coptisine, palmatine, and berberine, remained in their nascent form when measured in rat urine [24]. These results demonstrate that berberine and coptisine do not get metabolized in the blood and are excreted in their original form from the urine. This suggests that berberine and coptisine could reach the hepatic and liver cells. Therefore, the high-affinity interaction we demonstrated in our work should occur. The other alkaloids studied here would either fail to enter into blood or have no affinity for liver cells.

Previous work has found evidence that berberine affects glucose metabolism, leading to an increase in insulin secretion, suppression of adipogenesis, inhibition of mitochondrial function, and activation of the 5′ adenosine monophosphate-activated protein kinase (AMPK) pathway [25–28]. This antidiabetic and insulin sensitizing effect of berberine has been confirmed in a few relatively small, short-term clinical trials [29]. To date, very few reports on the lipid regulation by the other alkaloids derived from CAE have been published. In our experiments, coptisine had a superior lipid reducing effect compared with berberine. Several studies of the pharmacologic activity of coptisine have reported vasorelaxant action [30], a cardioprotective effect [31], an antidiabetic effect [32], and an antimicrobial effect [33]. This suggests that coptisine deserves to be further explored as an antihepatic steatosis agent. However, other alkaloids, including palmatine, jateorhizine, epiberberine, columbamine, and magnoflorine, also have a certain degree of lipid reduction activity. Our work demonstrated that the five other alkaloids had weak activity as antihepatic steatosis agents singularly and no observable synergistic effect.

The relationship between the structure and activity of the seven alkaloids in CAE is very interesting. All seven are derivatives of benzyl tetrahydroisoquinoline alkaloids. However, there are subtle differences between them due to substitution groups (Figure 1) that result in the affinity and activity differences observed in our experiments. Structural analysis revealed that berberine and coptisine possess a common methylenedioxy group at C2, C3, differing from the other alkaloids. The reports on the relationship between the structure and function have proven that when methylenedioxy groups were substituted in, an enhancement of the antibacterial activity of the alkaloids was seen. Meanwhile, there was an increased toxic effect when substituted with methoxyl groups [34, 35]. Berberine studies have also showed that methylenedioxy groups at C2, C3 are important groups for antimicrobial [33] and antifungal properties [36]. In addition, the methylenedioxy group plays an important role in hepatic mitochondrial-reduced glutathione (GSH) stimulatory activity as shown from schisandrin studies [37]. These results suggest that methylenedioxy could be a key active group in antihepatic steatosis. Moreover, besides a common methylenedioxy group at C2, C3, coptisine has another methylenedioxy group at C9, C10, while berberine has a methoxyl group at the C9, C10.

Lipid metabolism in hepatocytes is primarily regulated by the homeostasis of intracellular lipid within the cells. Fatty acid *β*-oxidation and VLDL equipment are both located within the cytoplasm. The nuclear receptors regulating lipogenesis, also located in the cytoplasm, include SREBP-1C, PPAR-*α*, and LXR [1, 2]. Therefore, antihepatic steatosis agents must display an affinity for the cell membrane or become intracellular to have an effect. Liver cell extraction with HPLC analysis is a method of screening for components with a high-affinity towards hepatocytes, suggesting that this method is suitable for screening for antihepatic steatosis studies. In order to increase the specificity of this method, *in vitro* FFA-induced hepatic steatosis of HepG2 liver cells was also used as an evaluative tool after liver cell extraction. Because the same type of cells was used in both the screening and evaluation, an agreement between the two methods was guaranteed. It believed that this method will be applicable and a strong tool for studying other multicomponential extracts from TCMs, such as flavonoids, saponins, and terpenes acid.

## Figures and Tables

**Table 1:** Intracellular TG content of hepatic steatosis cell model treated with alkaloids.

Sample group	Magnoflorine	Columbamine	Jateorhizine	Epiberberine	Coptisine	Palmatine	Berberine
Control group	37 ± 03	42 ± 12	57 ± 06	79 ± 10	39 ± 07	70 ± 14	47 ± 13
Model group	464 ± 34^##^	456 ± 32^##^	510 ± 15^##^	520 ± 31^##^	485 ± 29^##^	520 ± 11^##^	473 ± 12^##^
0.01 *μ*g/mL					424 ± 15		
0.04 *μ*g/mL					327 ± 12*		
0.2 *μ*g/mL	397 ± 22	438 ± 06	475 ± 07	516 ± 09	248 ± 09**	528 ± 15	390 ± 04*
1.0 *μ*g/mL	350 ± 19*	376 ± 23*	475 ± 01	468 ± 29	240 ± 03**	460 ± 06*	308 ± 08**
5.0 *μ*g/mL	336 ± 06*	316 ± 13**	509 ± 19	390 ± 13*	240 ± 15**	410 ± 27**	225 ± 12**
25.0 *μ*g/mL	276 ± 25**	234 ± 21**	541 ± 14	336 ± 11**	231 ± 06**	339 ± 24**	183 ± 03**

^∗^Indicates a significant difference compared with model group cells, and # indicates a significant difference compared with control group cells. Values were mean ± SD (*n* = 6) and expressed in *μ*mol/g protein.

*P < 0.05, **P < 0.01, ^##^ *P* < 0.01.
